# First-trimester predictive models for adverse pregnancy outcomes—a base for implementation of strategies to prevent cardiovascular disease development

**DOI:** 10.3389/fcell.2024.1461547

**Published:** 2024-09-04

**Authors:** Ilona Hromadnikova, Katerina Kotlabova, Ladislav Krofta

**Affiliations:** ^1^ Department of Molecular Biology and Cell Pathology, Third Faculty of Medicine, Charles University, Prague, Czechia; ^2^ Institute for the Care of the Mother and Child, Third Faculty of Medicine, Charles University, Prague, Czechia

**Keywords:** first-trimester screening, cardiovascular risk, miRNA, predictive models, preventive program, risk factors

## Abstract

**Introduction:**

This study aimed to establish efficient, cost-effective, and early predictive models for adverse pregnancy outcomes based on the combinations of a minimum number of miRNA biomarkers, whose altered expression was observed in specific pregnancy-related complications and selected maternal clinical characteristics.

**Methods:**

This retrospective study included singleton pregnancies with gestational hypertension (GH, n = 83), preeclampsia (PE, n = 66), HELLP syndrome (n = 14), fetal growth restriction (FGR, n = 82), small for gestational age (SGA, n = 37), gestational diabetes mellitus (GDM, n = 121), preterm birth in the absence of other complications (n = 106), late miscarriage (n = 34), stillbirth (n = 24), and 80 normal term pregnancies. MiRNA gene expression profiling was performed on the whole peripheral venous blood samples collected between 10 and 13 weeks of gestation using real-time reverse transcription polymerase chain reaction **(**RT-PCR).

**Results:**

Most pregnancies with adverse outcomes were identified using the proposed approach (the combinations of selected miRNAs and appropriate maternal clinical characteristics) (GH, 69.88%; PE, 83.33%; HELLP, 92.86%; FGR, 73.17%; SGA, 81.08%; GDM on therapy, 89.47%; and late miscarriage, 84.85%). In the case of stillbirth, no addition of maternal clinical characteristics to the predictive model was necessary because a high detection rate was achieved by a combination of miRNA biomarkers only [91.67% cases at 10.0% false positive rate (FPR)].

**Conclusion:**

The proposed models based on the combinations of selected cardiovascular disease-associated miRNAs and maternal clinical variables have a high predictive potential for identifying women at increased risk of adverse pregnancy outcomes; this can be incorporated into routine first-trimester screening programs. Preventive programs can be initiated based on these models to lower cardiovascular risk and prevent the development of metabolic/cardiovascular/cerebrovascular diseases because timely implementation of beneficial lifestyle strategies may reverse the dysregulation of miRNAs maintaining and controlling the cardiovascular system.

## 1 Introduction

MiRNAs are small non-coding RNAs (18–25 nucleotides) that regulate gene expression at the post-transcriptional level (Lai, 2002; Bartel, 2004). Increased miRNA expression results in the degradation of mRNAs or blockage of translation of potential target genes. Conversely, upregulation of potential target genes results from decreased miRNA levels. An altered miRNA expression profile usually contributes to the pathophysiology of the disease and may be used for the diagnosis and/or the assessment of prognosis of the disease (Piletič and Kunej, 2016; Wang et al., 2016; Condrat et al., 2020).

Recently, we observed an altered expression profile of miRNAs that play a role in homeostasis and maintenance of the cardiovascular system and the pathophysiology of cardiovascular and cerebrovascular diseases in women at risk of adverse pregnancy outcomes (Hromadnikova et al., 2022d; Hromadnikova et al., 2023a). Initially, we proposed early predictive models for gestational hypertension (GH) (Hromadnikova et al., 2022a), preeclampsia (PE) (Hromadnikova et al., 2022a), HELLP syndrome (Hromadnikova et al., 2023a), fetal growth restriction (FGR) (Hromadnikova et al., 2022b), small for gestational age (SGA) (Hromadnikova et al., 2022b), preterm delivery in the absence of other pregnancy-related complications (Hromadnikova et al., 2022c), gestational diabetes mellitus (GDM) (Hromadnikova et al., 2022d), miscarriage or stillbirth (Hromadnikova et al., 2023c) based only on miRNA biomarkers.

Afterwards, we identified multiple independent risk factors predisposing to the development of pregnancy-related complications such as maternal age and body mass index (BMI) at early stages of gestation, nulliparity, confirmed diagnosis of autoimmune disease, infertility treatment using assisted reproductive technology, presence of chronic hypertension, presence of thrombophilia gene mutations, history of pregnancy-related complications (PE, HELLP, SGA, FGR, and preterm birth) in previous pregnancy (ies), history of miscarriage (before 20 gestational weeks), and occurrence of diabetes mellitus in first-degree relatives (Hromadnikova et al., 2024; Hromadnikova et al., 2023a; Hromadnikova et al., 2023b; Hromadnikova et al., 2022e; Hromadnikova et al., 2022d; Hromadnikova et al., 2023c).

Subsequently, we involved these maternal clinical characteristics in miRNA-based predictive models, which increased the detection rate of pregnancies at high risk of adverse pregnancy outcomes (Hromadnikova et al., 2024; Hromadnikova et al., 2023a; Hromadnikova et al., 2023b; Hromadnikova et al., 2022e; Hromadnikova et al., 2022d; Hromadnikova et al., 2023c). In addition, we added first-trimester screening for PE and/or FGR and spontaneous preterm birth, both determined using the FMF algorithm (Tan et al., 2018), to the predictive models for GH, PE, HELLP syndrome, FGR, SGA, and GDM, as these two independent variables slightly increased the detection rates.

Currently, we focused on the development of efficient, cost-effective, early predictive models for identifying adverse pregnancy outcomes based on a selection of six miRNAs (miR-181a-5p, miR-20a-5p, miR-146a-5p, miR-574-3p, miR-1-3p, and miR-16-5p), whose altered expression was a common phenomenon shared between multiple pregnancy-related complications (Table 1). These miRNAs were combined with maternal clinical characteristics previously identified as the risk factors for a complicated gestational course (Table 2).

**TABLE 1:** MiRNA altered expression profile during early gestational stages - common sign of adverse pregnancy outcomes.

	GH	PE	HELLP	FGR	SGA	Pretem delivery (PPROM or PTB) No other complications	GDM on therapy	Pregnancy loss	
								Late miscarriage	Stillbirth
miR-181a-5p	+	+	+	+	+			+	+
miR-20a-5p		+		+	+		+		+
miR-146a-5p		+	+	+	+	+		+	+
miR-574-3p		+		+					+
miR-1-3p			+		+			+	+
miR-16-5p				+		+		+	+

GH, gestational hypertension; PE, preeclampsia; FGR, fetal growth restriction; SGA, small for gestational age; GDM, gestational diabetes mellitus; PPROM, preterm prelabor rupture of membranes; PTB, spontaneous preterm birth; HELLP, hemolysis, elevated liver enzymes and low platelets syndrome.

**TABLE 2:** Maternal clinical characteristics representing risk factors for adverse pregnancy outcomes involved in first-trimester predictive models.

Variables involved in prediction models	GH	PE	HELLP	FGR	SGA	Preterm delivery^a^	GDM	Late miscarriage
Maternal age at early gestational stages	+	+	+	+	+	+	++	+
BMI at early gestational stages	+	+	+	+	+	+	++	+
Nulliparity	+	+		+				
Confirmed diagnosis of autoimmune disease	+	+	+	+		+		+
Chronic hypertension in anamnesis				+				
Family history of diabetes mellitus (first-degree relatives only)							+	
Current pregnancy conceived after ART techniques (IVF/ICSI/other)	+	+	+	+	+	+	++	+
Trombophilia gene mutations			+				+	+
History of miscarriage (spontaneous loss of pregnancy before 20 weeks of gestation)							+	+
History of HELLP and/or PE		+	+					
History of SGA or FGR				+				
History of preterm birth						+		
Presence of non-autoimmune hypothyroidism								+
Presence of uterine fibroids or abnormal shaped womb								+
Predictive Model I-Number of Variables	**5**	**6**	**6**	**7**	**3**	**5**	**3**	**8**
Screen-positive for PE and/or FGR by FMF algorithm	+	+	+	+	+	+	+	+
Screen-positive for preterm birth by FMF algorithm	+	+	NA^b^	+	+	+	NA^c^	
Predictive Model II-Number of Variables (Model I + FMF screening results)	**7**	**8**	**7**	**9**	**5**	**7**	**7**	**9**

GH, gestational hypertension; PE, preeclampsia; HELLP, haemolysis, elevated liver enzymes and low platelets syndrome; FGR, fetal growth restriction; SGA, small-for-gestational-age; PTB, spontaneous preterm birth; PPROM, preterm prelabor rupture of membranes; GDM, gestational diabetes mellitus; BMI, body mass index; ART, assisted reproductive technology; IVF, *in vitro* fertilization; ICSI, intracytoplasmic sperm injection; FMF, Fetal Medicine Foundation. Bold values highlight the final number of variables used in Predictive Model I and Predictive Model II.

^a^ PPROM, or PTB, with no other complications; NA, not analysed in model.

^b^ low number of patients with appropriate data.

^c^ no occurrence of preterm birth in this group of pregnancies; ++, maternal clinical variables used in prediction model I for GDM.

## 2 Materials and methods

### 2.1 Patients cohort

This study included pregnancies diagnosed with gestational hypertension (n = 83), preeclampsia (n = 66), HELLP syndrome (n = 14), fetal growth restriction (n = 82), small for gestational age (n = 37), preterm birth [spontaneous preterm birth (PTB) or preterm prelabor rupture of membranes (PPROM)] in the absence of other pregnancy-related complications (n = 106), gestational diabetes mellitus requiring administration of appropriate therapy (n = 20), late miscarriage (n = 34), and stillbirth (n = 24) together with reference group (normal term pregnancies, n = 80).

#### 2.1.1 Inclusion and exclusion criteria

- Singleton pregnancies of Caucasian descent only undergoing the first-trimester screening at 10–13 weeks of gestation
- Pregnancies with confirmed adverse obstetric outcomes. The diagnoses were assessed using appropriate guidelines (ACOG Committee on Practice Bulletins--Obstetrics, 2002; ACOG Committee on Practice Bulletins—Gynecology, 2018; ACOG Committee on Practice Bulletins--Obstetrics, 2020; American College of Obstetricians and Gynecologists et al., 2020; ACOG Committee on Practice Bulletins--Obstetrics, 2021; American Diabetes Association, 2009; International Association of Diabetes and Pregnancy Study Groups Consensus Panel, 2010; Martin et al., 1991; Martin et al., 2006; Weinstein, 1982; Audibert et al., 1996; Moutquin et al., 1996; Sibai, 2004; Barton and Sibai, 2004; Romero et al., 2006; Goldenberg et al., 2008; Leeners et al., 2011; Malmström and Merken, 2018).
- Only pregnancies with complete medical records that had been followed up and delivered at the Institute for the Care of Mother and Child, Prague, Czech Republic
- PE: pregnancies with the onset of PE with or without FGR irrespective of the severity of the disease and gestational age of the onset of the disease
- HELLP syndrome: pregnancies with the onset of HELLP syndrome with or without PE with no sign of SGA or FGR
- SGA or FGR: only cases without PE regardless of the gestational age of the onset of the disease
- Preterm birth: PTB or PPROM occurring before 37 gestational weeks in the absence of other pregnancy-related complications (GH, PE, HELLP, FGR, SGA, or GDM)
- GDM: Patients newly diagnosed with diabetes mellitus during early gestation, patients with the occurrence of chronic hypertension, and those ones carrying growth restricted or SGA fetuses, fetuses with anomalies or chromosomal abnormalities were intentionally excluded from the study. Likewise, patients demonstrating concurrently other pregnancy-related complications such as GH, PE, HELLP syndrome, *in utero* infections, PTB, PPROM, fetal demise *in utero* or stillbirth were also excluded from the study.
- Pregnancy losses: late miscarriage occurring between 13 and 20 weeks of gestation or stillbirth occurring after 20 weeks of gestation, both explained and unexplained causes were included in the study
- Selected maternal-age-matched normal term pregnancies
- Selected gestational-age-matched at sampling (weeks) normal term pregnancies

The selection of maternal-age-matched, and gestational-age-matched at sampling (weeks) normal term pregnancies ensured the homogeneity and comparability between the studied groups.

Pilot and validation studies were performed. Sample size calculation was used to calculate the minimal required sample size of subjects for analyses.

All procedures were in accordance with the ethical standards of the responsible committee on human experimentation (institutional and national) and the Helsinki Declaration of 1964 and its later amendments. All the included patients provided informed consent for participation in the study. The Ethics Committee of the Third Faculty of Medicine, Charles University, granted initial approval for this study (Implication of placental-specific miRNAs in maternal circulation for diagnosis and prediction of pregnancy-related complications, date of approval: 7 April 2011). Ongoing approval for the study was obtained from the Ethics Committee of the Third Faculty of Medicine, Charles University (Long-term monitoring of complex cardiovascular profiles in mother, fetus, and offspring descending from pregnancy-related complications, date of approval: 27 March 2014) and the Ethics Committee of the Institute for the Care of the Mother and Child, Charles University (Long-term monitoring of complex cardiovascular profiles in mother, fetus, and offspring descending from pregnancy-related complications, date of approval: 28 May 2015, number of approval: 1/4/2015). Informed consent is a complex process as it involves attaining consent for collecting peripheral blood samples at the beginning of pregnancy. In addition, it also includes gaining consent for collecting peripheral blood samples at the onset of pregnancy-related complications and collecting placental samples during childbirth in case of the onset of pregnancy-related complications.

### 2.2 Collection and processing of samples

Collection and processing of samples, reverse transcription (RT), and real-time PCR analyses were performed as previously described (Hromadnikova et al., 2022a; Hromadnikova et al., 2022b; Hromadnikova et al., 2022c; Hromadnikova et al., 2022d; Hromadnikova et al., 2022e; Hromadnikova et al., 2023a; Hromadnikova et al., 2023b; Hromadnikova et al., 2023c; Hromadnikova et al., 2024).

Briefly, total RNA enriched for small RNAs was isolated from whole peripheral venous blood (EDTA) using a mirVana miRNA isolation kit (Ambion, Austin, United States of America). mRNAs of miRNAs of interest were reverse transcribed into complementary DNA (cDNA) using miRNA-specific stem loop primers and TaqMan MicroRNA Reverse Transcription Kit (Applied Biosystems, Branchburg, United States of America). Reverse transcription was performed in a total reaction volume of 10 µL.

Subsequently, 3 µL of cDNA was mixed in a total reaction volume of 15 µL with specific primers, TaqMan MGB probes (the components of TaqMan MicroRNA Assays), and the components of the TaqMan Universal PCR Master Mix (Applied Biosystems, Branchburg, United States of America). Real-time RT-qPCR was performed on a 7,500 Real-Time PCR System under standard TaqMan PCR conditions described in the TaqMan guidelines. The miRNA gene expression was determined using the comparative Ct method (Livak and Schmittgen, 2001). The normalization factor (Vandesompele et al., 2002) (geometric mean of Ct values of selected endogenous controls: RNU58A and RNU38B) was used to normalize the miRNA gene expression data.

### 2.3 Criteria for the MiRNA selection

In total, 29 miRNAs were screened at early stages of gestation in pregnancies at risk of adverse pregnancy outcomes. The set involved the following miRNAs: miR-1-3p, miR-16-5p, miR-17-5p, miR-20a-5p, miR-20b-5p, miR-21-5p, miR-23a-3p, miR-24-3p, miR-26a-5p, miR-29a-3p, miR-92a-3p, miR-100-5p, miR-103a-3p, miR-125b-5p, miR-126-3p, miR-130b-3p, miR-133a-3p, miR-143-3p, miR-145-5p, miR-146a-5p, miR-155-5p, miR-181a-5p, miR-195-5p, miR-199a-5p, miR-210-3p, miR-221-3p, miR-342-3p, miR-499a-5p, and miR-574-3p. Only the most frequently dysregulated miRNAs (miR-181a-5p, miR-20a-5p, miR-146a-5p, miR-574-3p, miR-1-3p, and miR-16-5p) were selected for the cost-effective early predictive models for adverse obstetric outcomes (Table 1; Table 3). Other miRNAs showed either no altered expression or were dysregulated in no more than two pregnancy-related complications.

**TABLE 3:** Characteristics of selected MiRNAs.

Assay name (the manufacturer)	Assay ID (the manufacturer)	miRBase ID	NCBI Location Chromosome	Mature miRNA sequence
hsa-miR-1	002222	hsa-miR-1-3p	Chr20: 62554306–62554376 [+]	5′-UGG​AAU​GUA​AAG​AAG​UAU​GUA​U-3′
hsa-miR-16	000391	hsa-miR-16-5p	Chr13: 50048973–50049061 [-]	5′-UAG​CAG​CAC​GUA​AAU​AUU​GGC​G- 3′
hsa-miR-20a	000580	hsa-miR-20a-5p	Chr13: 91351065–91351135 [+]	5′-UAA​AGU​GCU​UAU​AGU​GCA​GGU​AG-3′
hsa-miR-146a	000468	hsa-miR-146a-5p	Chr5: 160485352–160485450 [+]	5′-UGA​GAA​CUG​AAU​UCC​AUG​GGU​U-3′
hsa-miR-181a	000480	hsa-miR-181a-5p	Chr1: 198859044–198859153 [-]	5′-AAC​AUU​CAA​CGC​UGU​CGG​UGA​GU-3′
hsa-miR-574-3p	002349	hsa-miR-574-3p	Chr4: 38868032–38868127 [+]	5′-CAC​GCU​CAU​GCA​CAC​ACC​CAC​A-3′

### 2.4 Statistical analysis

Predictive models for adverse pregnancy outcomes were constructed using logistic regression and receiver operating characteristic (ROC) curve analyses (MedCalc Software bvba, Ostend, Belgium). ROC curves displayed the areas under the curves (AUC), the cut-off points associated with sensitivities, specificities, positive and negative likelihood ratios (LR+, LR-), and sensitivities at 10.0% false positive rate (FPR) (MedCalc Software bvba, Ostend, Belgium). Initially, all independent variables (selected miRNAs and maternal clinical characteristics) and dependent variables (diagnoses, for example preeclampsia – 1, normal term pregnancies - 0) were entered into the logistic regression models for particular pregnancy-related complications. Subsequent ROC curve analyses were applied (MedCalc Software bvba, Ostend, Belgium), where the predictive probabilities gained from logistic regression analyses were saved and next used as the new variables and the diagnoses (for example preeclampsia – 1, normal term pregnancies - 0) acted as the classification variables in ROC curve analyses.

### 2.5 Analysis of MiRNA-target interactions

The miRWalk database (http://mirwalk.umm.uni-heidelberg.de/) and disease ontology module (http://mirwalk.umm.uni-heidelberg.de/diseases/) were used to provide information on the predicted and/or validated targets of miRNAs. Pregnancy-related complications, such as preeclampsia, HELLP syndrome, placental insufficiency, and GDM were available in the miRWalk database. The only common targets associated with pregnancy-related complications, cardiovascular risk factors (obesity, hypertension, atherosclerosis, prediabetes syndrome, and diabetes mellitus), and cardiovascular and cerebrovascular diseases (myocardial infarction, cerebral infarction, systolic and diastolic heart failure, and heart, cardiovascular, and cerebrovascular diseases as a whole) were reported.

## 3 Results

### 3.1 The cost-effective first-trimester predictive models for adverse pregnancy outcomes

The cost-effective first-trimester predictive models for adverse pregnancy outcomes were based on the combinations of a minimum number of miRNA biomarkers with jointly altered expression during the early gestational stages. In addition, maternal clinical characteristics identified as the risk factors for adverse pregnancy outcomes were added into the predictive models.

Several miRNAs of the six joint miRNAs were dysregulated at the early gestational stages in pregnancies with various adverse pregnancy outcomes (GH: 1 miRNA; PE: 4 miRNAs; HELLP syndrome: 3 miRNAs; FGR: 5 miRNAs; SGA: 4 miRNAs; GDM on therapy: 1 miRNA; late miscarriage: 4 miRNAs; stillbirth: six miRNAs; and preterm delivery in the absence of the above-mentioned pregnancy-related complications: two miRNAs).

The combinations of these miRNAs correctly predicted the occurrence of various adverse pregnancy outcomes in a portion of cases at 10.0% FPR (GH: 22.89% cases; PE: 48.48% cases; HELLP syndrome: 57.14% cases; FGR: 37.80% cases; SGA: 75.68% cases; GDM on therapy: 20.0% cases; late miscarriage: 52.94% cases; stillbirth: 91.67% cases; and preterm delivery in the absence of the above-mentioned pregnancy-related complications: 27.36% cases) (Table 4).

**TABLE 4:** Predictive models for adverse pregnancy outcomes based on the combinations of MiRNA biomarkers with jointly altered expression during early gestational stages and maternal clinical variables representing risk factors for adverse pregnancy outcomes.

	AUC	95% CI	*p*- value	Sensitivity	Criterion	Youden index J	Youden index associated Criterion	Sensitivity	95%CI	Specificity	95%CI	+LR	95%CI	-LR	95%CI
				(At 10% FPR)											
Predictive Model for GH (Hromadnikova et al. 2024)															
1 miRNA only (miR-181a-5p)	0.649	0.570–0.722	<0.001	22.89%	>0.5216	0.2645	>0.2618	61.45%	50.1–71.9	65.00%	53.5–75.3	1.76	1.2–2.5	0.59	0.4–0.8
1 miRNA +5 maternal clinical characteristics	0.858	0.794–0.907	<0.001	62.65%	>0.6399	0.5577	>0.4314	79.52%	69.2–87.6	76.25%	65.4–85.1	3.35	2.2–5.0	0.27	0.2–0.4
1 miRNA +7 maternal clinical characteristics	0.894	0.837–0.937	<0.001	69.88%	>0.5692	0.6627	>0.6955	66.27%	55.1–76.3	100.0%	95.5–100.0	-	-	0.34	0.2–0.5
Predictive Model for PE															
4 miRNAs only (miR-181a-5p, miR-20a-5p, miR-146a-5p, and miR-574-3p)	0.715	0.634–0.786	<0.001	48.48%	>0.5444	0.3973	>0.5504	48.48%	36.0–61.1	91.25%	82.8–96.4	5.54	2.6–11.7	0.56	0.4–0.7
4 miRNAs +6 maternal clinical characteristics	0.902	0.842–0.945	<0.001	78.79%	>0.4562	0.7004	>0.4657	78.79%	67.0–87.9	91.25%	82.8–96.4	9.00	4.4–18.5	0.23	0.1–0.4
4 miRNAs +8 maternal clinical characteristics	0.934	0.880–0.968	<0.001	83.33%	>0.3707	0.7905	>0.5331	80.30%	68.7–89.1	98.75%	93.2–100.0	64.24	9.1–452.2	0.20	0.1–0.3
Predictive Model for HELLP															
3 miRNAs only (miR-181a-5p, miR-146a-5p, and miR-1-3p)	0.895	0.814–0.949	<0.001	57.14%	>0.1862	0.6446	>0.1161	85.71%	57.2–98.2	78.75%	68.2–87.1	4.03	2.5–6.5	0.18	0.05–0.7
3 miRNAs +6 maternal clinical characteristics	0.970	0.912–0.994	<0.001	85.71%	>0.1106	0.8161	>0.0855	92.86%	66.1–99.8	88.75%	79.7–94.7	8.25	4.4–15.5	0.008	0.01–0.5
3 miRNAs +7 maternal clinical characteristics	0.969	0.911–0.994	<0.001	92.86%	>0.1116	0.8786	>0.1704	92.86%	66.1–99.8	95.00%	87.7–98.6	18.57	7.1–48.8	0.075	0.01–0.5
Predictive Model for FGR															
5 miRNAs only (miR-181a-5p, miR-20a-5p, miR-146a-5p, miR-574-3p, and miR-16-5p)	0.680	0.602–0.751	<0.001	37.80%	>0.6185	0.3409	>0.6919	36.59%	26.2–48.0	97.50%	91.3–99.7	14.63	3.6–59.2	0.65	0.5–0.8
5 miRNAs +7 maternal clinical characteristics	0.815	0.747–0.872	<0.001	58.54%	>0.5995	0.5326	>0.4896	69.51%	58.4–79.2	83.75%	73.8–91.1	4.28	2.5–7.2	0.36	0.3–0.5
5 miRNAs +9 maternal clinical characteristics	0.860	0.797–0.909	<0.001	73.17%	>0.5002	0.6701	>0.6066	69.51%	58.4–79.2	97.50%	91.3–99.7	27.80	7.0–110.1	0.31	0.2–0.4
Predictive Model for SGA (Hromadnikova et al., 2023b)															
4 miRNAs only (miR-181a-5p, miR-20a-5p, miR-146a-5p, and miR-1-3p)	0.868	0.792–0.923	<0.001	75.68%	>0.3664	0.6568	>0.3664	75.68%	58.8–88.2	90.00%	81.2–95.6	7.57	3.8–15.0	0.27	0.2–0.5
4 miRNAs +3 maternal clinical characteristics	0.870	0.795–0.925	<0.001	70.27%	>0.3844	0.6443	>0.3309	75.68%	58.8–88.2	88.75%	79.7–94.7	6.73	3.5–12.8	0.27	0.2–0.5
4 miRNAs +5 maternal clinical characteristics	0.922	0.858–0.964	<0.001	81.08%	>0.3109	0.7253	>0.2844	83.78%	68.0–93.8	88.75%	79.7–94.7	7.45	4.0–14.0	0.18	0.09–0.4
Predictive Model for GDM on therapy															
1 miRNA only (miR-20a-5p)	0.709	0.610–0.796	<0.001	20.00%	>0.2865	0.3625	>0.1343	100.00%	83.2–100.0	36.25%	25.8–47.8	1.57	1.3–1.9	0	-
1 miRNA +3 maternal clinical characteristics	0.949	0.886–0.983	<0.001	78.95%	>0.2605	0.7447	>0.1674	89.47%	66.9–98.7	85.00%	75.3–92.0	5.96	3.5–10.3	0.12	0.03–0.5
1 miRNA +7 maternal clinical characteristics	0.957	0.896–0.987	<0.001	89.47%	>0.2039	0.7947	>0.2039	89.47%	66.9–98.7	90.00%	81.2–95.6	8.95	4.6–17.6	0.12	0.03–0.4
Predictive Model for Late Miscarriage															
4 miRNAs only (miR-181a-5p, miR-146a-5p, miR-16-5p, and miR-1-3p)	0.828	0.746–0.892	<0.001	52.94%	>0.3683	0.5140	>0.2598	67.65%	49.5–82.6	83.75%	73.8–91.1	4.16	2.4–7.2	0.39	0.2–0.6
4 miRNAs +8 maternal clinical characteristics	0.936	0.874–0.973	<0.001	84.85%	>0.2005	0.7807	>0.3892	81.82%	64.5–93.0	96.25%	89.4–99.2	21.82	7.1–67.0	0.19	0.09–0.4
4 miRNAs +9 maternal clinical characteristics	0.935	0.873–0.973	<0.001	84.85%	>0.1967	0.7735	>0.2471	84.85%	68.1–94.9	92.50%	84.4–97.2	11.31	5.2–24.8	0.16	0.07–0.4
Predictive Model for Stillbirth															
6 miRNAs only (miR-181a-5p, miR-20a-5p, miR-146a-5p, miR-574-3p, miR-16-5p, and miR-1-3p)	0.967	0.912–0.992	<0.001	91.67%	>0.0602	0.9167	>0.2737	91.67%%	73.0–99.0	100.00%	95.5–100.0	-	-	0.083	0.02–0.3
Predictive Model for Preterm Delivery (PTB or PPROM) in the Absence of Other Pregnancy-Related Complications															
2 miRNAs only (miR-16-5p and miR-146a-5p)	0.631	0.557–0.700	<0.001	27.36%	>0.6804	0.2212	>0.6570	39.62%	30.3–49.6	82.50%	72.4–90.1	2.26	1.3–3.8	0.73	0.6–0.9
2 miRNAs +5 maternal clinical characteristics	0.740	0.670–0.801	<0.001	45.28%	>0.5930	0.3934	>0.6010	44.34%	34.7–54.3	95.00%	87.7–98.6	8.87	3.3–23.6	0.59	0.5–0.7
2 miRNAs +7 maternal clinical characteristics	0.766	0.698–0.825	<0.001	51.89%	>0.5804	0.4252	>0.5769	53.77%	43.8–63.5	88.75%	79.7–94.7	4.78	2.5–9.1	0.52	0.4–0.6

GH, gestational hypertension; PE, preeclampsia; HELLP, haemolysis, elevated liver enzymes and low platelets syndrome; FGR, fetal growth restriction; SGA, small-for-gestational-age; GDM, gestational diabetes mellitus; PTB, spontaneous preterm birth; PPROM, preterm prelabor rupture of membranes.

Predictive models based on the combinations of these miRNAs and selected maternal clinical characteristics identified as the risk factors for appropriate adverse pregnancy outcomes in our previous studies showed higher detection rates at 10.0% FPR (GH, 62.65%; PE, 78.79%; HELLP syndrome, 85.71%; FGR, 58.54%; SGA, 70.27%; GDM on therapy, 78.95%; late miscarriage, 84.85%; and preterm delivery in the absence of the above-mentioned pregnancy-related complications, 45.28%) (Hromadnikova et al., 2022d; Hromadnikova et al., 2022e; Hromadnikova et al., 2023a; Hromadnikova et al., 2023b; Hromadnikova et al., 2023c; Hromadnikova et al., 2024) (Table 4). In the case of stillbirth, maternal clinical characteristics need not be added to the predictive model because the detection rate of cases was high only when using a combination of appropriate miRNAs.

More advanced predictive models, which included the results of first-trimester screening for PE and/or FGR and spontaneous preterm birth using the FMF algorithm, increased the detection rates of various adverse pregnancy outcomes at 10.0% FPR (GH: 69.88% cases; PE: 83.33% cases; HELLP syndrome: 92.86% cases; FGR: 73.17% cases; SGA: 81.08% cases; GDM on therapy: 89.47% cases; and preterm delivery in the absence of the above-mentioned pregnancy-related complications: 51.89% cases). In the case of late miscarriage, the detection rate remained the same at a FPR of 10.0% (84.85%) (Table 4).

### 3.2 Mutual comparison of individual first-trimester predictive models

Only one of six joint miRNAs (miR-181a-5p) was dysregulated at the early gestational stages in pregnancies developing GH. MiR-181a-5p was upregulated in 22.89% of cases with 10.0% FPR (Hromadnikova et al., 2022a). A predictive model based on a combination of the first-trimester expression profile of miR-181a-5p and five maternal clinical characteristics (maternal age and BMI at early gestational stages, nulliparity, confirmed diagnosis of autoimmune disease, and infertility treatment using assisted reproductive technology) reached a detection rate of 62.65% for GH cases at 10.0% FPR (Hromadnikova et al., 2024). A more advanced GH predictive model based on the combination of the first-trimester expression profile of miR-181a-5p and seven maternal clinical characteristics (adding the results gained from the first-trimester screening for PE and/or FGR and spontaneous preterm birth, both using the FMF algorithm) slightly increased the detection rate to 69.88% cases at 10.0% FPR (Hromadnikova et al., 2024). The predictive power for GH can only be improved using this approach.

Previously demonstrated PE predictive models based on the combinations of only six miRNAs (AUC 0.730, *p* < 0.001) (Hromadnikova et al., 2022a) or only eight miRNAs (AUC 0.815, *p* < 0.001) (Hromadnikova, 2023a) reached detection rates of 48.48% and 53.03%, respectively, at 10.0% FPR. Expanding the models based on miRNA expression profiles for the same selected maternal clinical characteristics representing risk factors for PE increased the predictive power significantly: six miRNAs +6 clinical variables (78.79% cases at 10.0% FPR, AUC 0.903, *p* < 0.001), eight miRNAs +6 clinical variables (77.27% cases at 10.0% FPR, AUC 0.931, *p* < 0.001), six miRNAs +8 clinical variables (84.85% cases at 10.0% FPR, AUC 0.939, *p* < 0.001), eight miRNAs +8 clinical variables (84.85% cases at 10.0% FPR, AUC 0.950, *p* < 0.001) (Hromadnikova et al., 2024). The PE predictive model based on four out of six miRNAs common to adverse pregnancy outcomes and the same maternal clinical characteristics (six variables or eight variables) reached a similar detection power (78.79% cases at 10.0% FPR, AUC 0.902, *p* < 0.001; 83.33% cases at 10.0% FPR, AUC 0.934, *p* < 0.001) as the similar models with a higher number of miRNA biomarkers and may be considered as the most cost-effective first-trimester predictive model for PE irrespective of disease severity and time of disease onset.

The HELLP syndrome predictive model previously demonstrated by our group based on the combination of six miRNAs (AUC 0.903, *p* < 0.001) (Hromadnikova et al., 2023a; Hromadnikova, 2022f) reached a detection rate of 78.57% at 10.0% FPR. When this model was expanded for the same selected maternal clinical characteristics representing risk factors for HELLP syndrome, the predictive power significantly increased: six miRNAs +6 clinical variables (85.71% cases at 10.0% FPR, AUC 0.979, *p* < 0.001) and six miRNAs +7 clinical variables (92.86% cases at 10.0% FPR, AUC 0.975, *p* < 0.001) (Hromadnikova et al., 2023a; Hromadnikova, 2022f). The HELLP syndrome predictive model based on three out of six miRNAs common to adverse pregnancy outcomes and the same maternal clinical characteristics (six variables or seven variables) reached similar detection power (85.71% cases at 10.0% FPR, AUC 0.970, *p* < 0.001; 92.86% cases at 10.0% FPR, AUC 0.969, *p* < 0.001) as similar models with six miRNA biomarkers and may be considered the most cost-effective first-trimester predictive model for HELLP syndrome.

Previously demonstrated FGR predictive models by our group based on the combinations of only seven miRNAs (AUC 0.725, *p* < 0.001) (Hromadnikova et al., 2022b) or 10 miRNAs (AUC 0.774, *p* < 0.001) (Hromadnikova, 2023a) reached a detection rate of 42.68% cases and 40.24% cases at 10.0% FPR. With the expansion with the same selected maternal clinical characteristics representing risk factors for FGR the models showed significantly increased predictive power: seven miRNAs +7 clinical variables (64.63% cases at 10.0% FPR, AUC 0.840, *p* < 0.001), 10 miRNAs +7 clinical variables (65.85% cases at 10.0% FPR, AUC 0.855, *p* < 0.001), seven miRNAs +9 clinical variables (74.39% cases at 10.0% FPR, AUC 0.887, *p* < 0.001), 10 miRNAs +9 clinical variables (78.05% cases at 10.0% FPR, AUC 0.896, *p* < 0.001) (Hromadnikova et al., 2023b). The FGR predictive models based on five out of six miRNAs common to adverse pregnancy outcomes and the same maternal clinical characteristics (seven variables or nine variables) reached a slightly lower detection power (58.54% cases at 10.0% FPR, AUC 0.815, *p* < 0.001; 73.17% cases at 10.0% FPR, AUC 0.860, *p* < 0.001) than similar models with a higher number of miRNA biomarkers. However, it may still be considered the most cost-effective first-trimester predictive models for FGR, irrespective of disease severity and time of disease onset.

Similarly, the most cost-effective first-trimester predictive model for SGA, which had already been presented, is based on the combination of four out of six miRNAs common to adverse pregnancy outcomes and five maternal clinical characteristics (81.08% cases at 10.0% FPR, AUC 0.922, *p* < 0.001) (Hromadnikova et al., 2023b). Another SGA predictive model containing eight miRNAs and five maternal clinical characteristics showed a slightly higher detection rate (89.19% cases at 10.0% FPR, AUC 0.956, *p* < 0.001) (Hromadnikova et al., 2023b). The combination of only four miRNAs (75.68% cases at 10.0% FPR, AUC 0.868, *p* < 0.001) (Hromadnikova et al., 2022b) or the combination of only eight miRNAs (83.78% cases at 10.0% FPR, AUC 0.926, *p* < 0.001) (Hromadnikova, 2023a) substantially impacted the SGA detection rate. The implementation of maternal clinical variables slightly increased the SGA detection rate.

A previously demonstrated predictive model for GDM requiring the administration of appropriate therapy by our group based on the combination of only three miRNAs (AUC 0.731, *p* < 0.001) (Hromadnikova et al., 2022d; Hromadnikova 2023b) reached a detection rate of 30.0% cases at 10.0% FPR. When this model was extended to the same selected maternal clinical characteristics representing risk factors for GDM, the predictive power was significantly increased: 3 miRNAs +3 clinical variables (78.95% cases at 10.0% FPR, AUC 0.949, *p* < 0.001) and 3 miRNAs +7 clinical variables (89.47% cases at 10.0% FPR, AUC 0.957, *p* < 0.001) (Hromadnikova et al., 2022d). The predictive model for GDM requiring administration of appropriate therapy based on 1 out of six miRNAs common to adverse pregnancy outcomes and the same maternal clinical characteristics (3 variables or seven variables) reached the same detection power (78.95% cases at 10.0% FPR, AUC 0.949, *p* < 0.001; 89.47% cases at 10.0% FPR, AUC 0.957, *p* < 0.001) as the similar models with a higher number of miRNA biomarkers and may be considered as the most cost-effective first-trimester predictive model for GDM requiring administration of appropriate therapy.

A previously demonstrated predictive model for late miscarriage by our group, based on the combination of only six miRNAs (AUC 0.941, *p* < 0.001) (Hromadnikova et al., 2023c), reached a detection rate of 79.41% at 10.0% FPR. Four of these miRNAs, dysregulated at early gestational stages in pregnancies affected by late miscarriage, were common to adverse pregnancy outcomes. The combination of only these four miRNAs was insufficient to predict the occurrence of late miscarriage (52.94% cases at 10.0% FPR, AUC 0.828, *p* < 0.001). The predictive model based on four miRNAs common to adverse pregnancy outcomes was further expanded to include maternal clinical characteristics (maternal age and BMI at early gestational stages, confirmed diagnosis of autoimmune disease, infertility treatment using assisted reproductive technology, presence of non-autoimmune hypothyroidism, presence of uterine fibroids or abnormal-shaped womb, history of miscarriage(s) in previous gestation(s), and presence of thrombophilia gene mutations) to increase the detection power of late miscarriage. Since the predictive power for late miscarriage significantly increased, this model can also be utilized as a cost-effective model (84.85% cases at 10.0% FPR, AUC 0.936, *p* < 0.001). Alternatively, this model may be extended to the results of first-trimester screening for PE and/or FGR using the FMF algorithm; however, the detection rate of pregnancies with late miscarriage remained the same as that of the model without this variable (84.85% cases at 10.0% FPR, AUC 0.935, *p* < 0.001).

Predictive models based on the combinations of only two miRNAs common to adverse pregnancy outcomes (91.67% cases at 10.0% FPR, AUC 0.951, *p* < 0.001) (Hromadnikova, 2022f; Hromadnikova et al., 2023a) or six miRNAs commonly associated with adverse pregnancy outcomes (91.67% cases at 10.0% FPR, AUC 0.967, *p* < 0.001) were sufficient to predict the later occurrence of stillbirth cost-effectively. Maternal clinical characteristics were not included in the stillbirth predictive models. A previously introduced predictive model for stillbirth based on a combination of 11 dysregulated miRNAs at the early gestational stages achieved a slightly higher detection power (95.83% cases at 10.0% FPR, AUC 0.986, *p* < 0.001) (Hromadnikova, 2022f; Hromadnikova et al., 2023a).

Previously demonstrated predictive models for preterm delivery (PPROM or PTB) in the absence of other pregnancy-related complications by our group, based on the combinations of six miRNAs (AUC 0.812, *p* < 0.001) or 12 miRNAs (AUC 0.818, *p* < 0.001) (Hromadnikova et al., 2022c; Hromadnikova, 2023a), reached a detection rate of 52.83% at 10.0% FPR. Extension of the models based on miRNA expression profiles for the same selected maternal clinical characteristics representing risk factors for preterm delivery in the absence of other pregnancy-related complications increased the predictive power significantly: six miRNAs +5 clinical variables (69.81% cases at 10.0% FPR, AUC 0.874, *p* < 0.001), 12 miRNAs +5 clinical variables (66.98% cases at 10.0% FPR, AUC 0.877, *p* < 0.001), six miRNAs +7 clinical variables (71.70% cases at 10.0% FPR, AUC 0.879, *p* < 0.001), 12 miRNAs +7 clinical variables (73.58% cases at 10.0% FPR, AUC 0.887, *p* < 0.001) (Hromadnikova et al., 2022e). The predictive models based on two out of six miRNAs common to adverse pregnancy outcomes and the same maternal clinical characteristics (five variables or seven variables) reached significantly lower detection power (45.28% cases at 10.0% FPR, AUC 0.740, *p* < 0.001; 51.89% cases at 10.0% FPR, AUC 0.766, *p* < 0.001) and cannot be considered as optimal cost-effective first-trimester predictive models for preterm delivery in the absence of other pregnancy-related complications.

### 3.3 Analysis of MiRNA-target interactions

**FIGURE 1:**
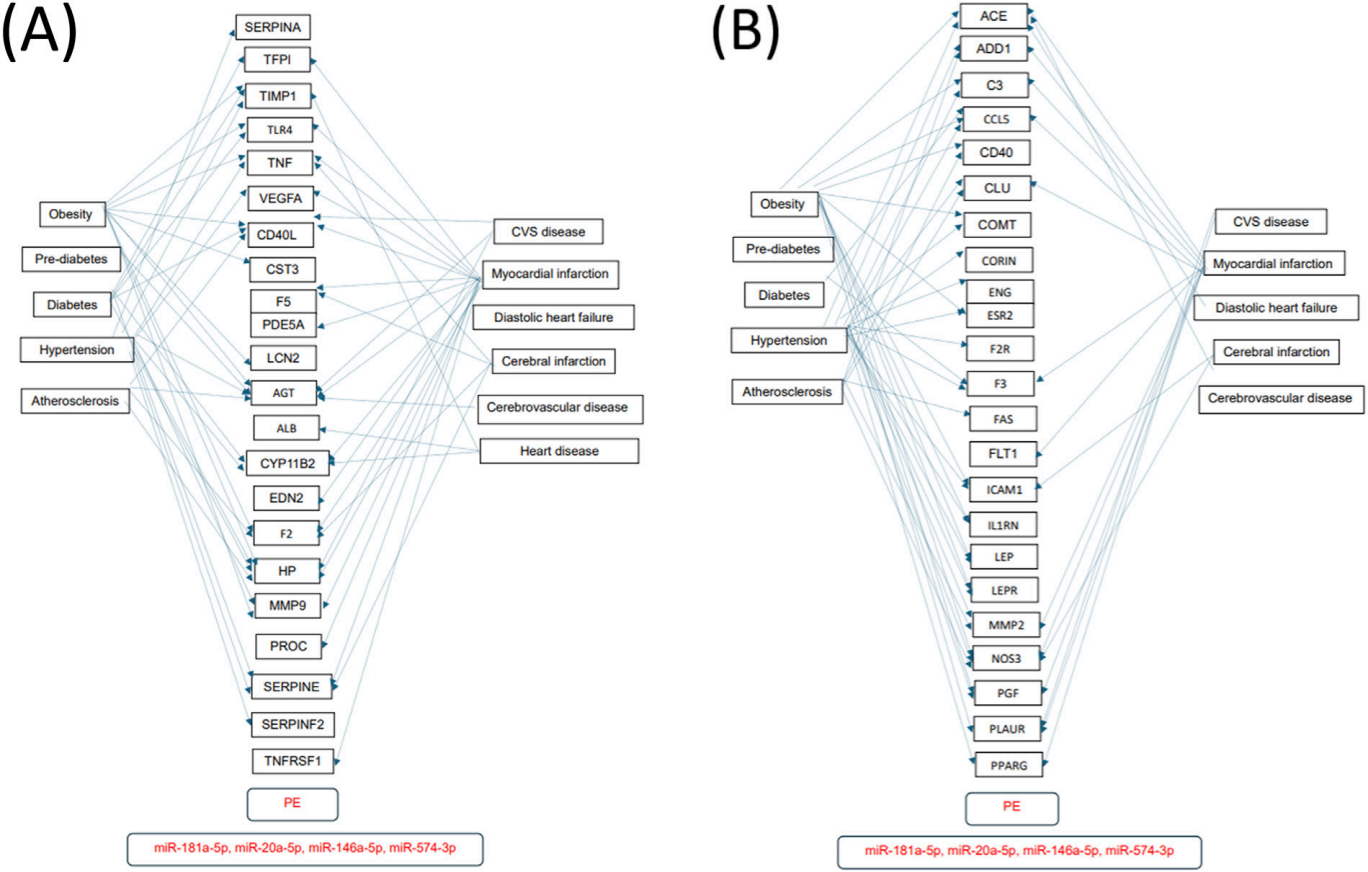
MiRNA-target interactions–Common targets of preeclampsia, cardiovascular risk factors, cardiovascular and cerebrovascular diseases. Search for interactions between miR-20a-5p, miR-146a-5p, miR-181a-5p, and miR-574-3p and genes using the miRWalk database and disease ontology module revealed numerous common targets **(A,B)** associated with preeclampsia, cardiovascular risk factors, cardiovascular and cerebrovascular diseases.

**FIGURE 2:**
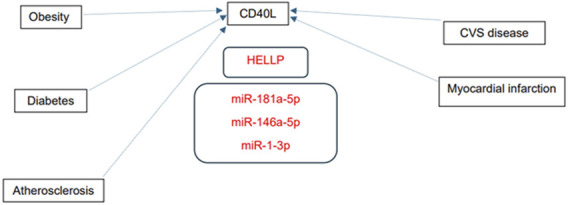
MiRNA-target interactions–Common targets of HELLP syndrome, cardiovascular risk factors and cardiovascular diseases. Search for interactions between miR-1-3p, miR-146a-5p, and miR-181a-5p and genes using the miRWalk database and disease ontology module revealed one common target (CD40LG, gene encoding CD40 ligand) associated with HELLP syndrome, cardiovascular risk factors and cardiovascular diseases.

**FIGURE 3:**
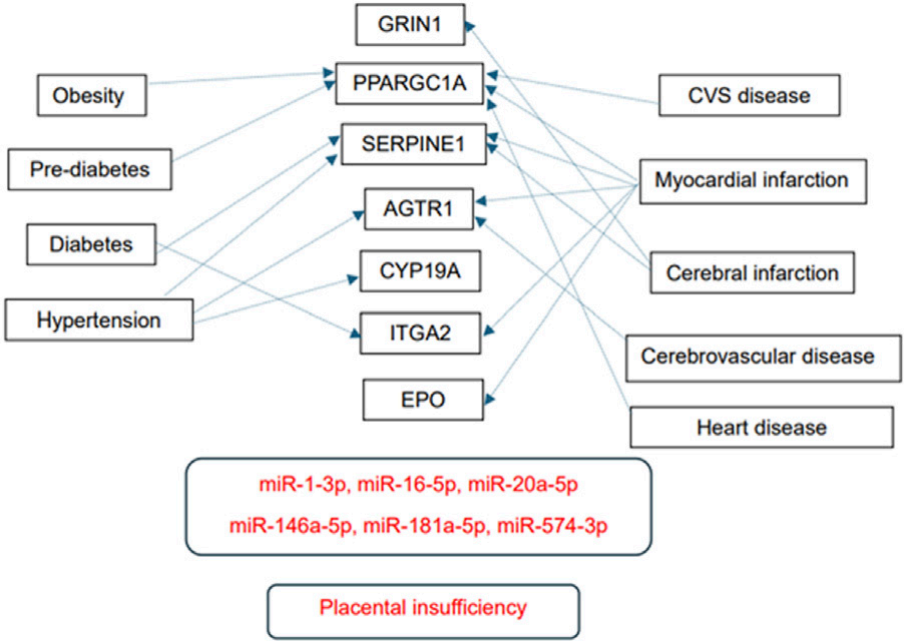
MiRNA-target interactions–Common targets of placental insufficiency, cardiovascular risk factors, cardiovascular and cerebrovascular diseases. Search for interactions between miR-1-3p, miR-16-5p, miR-20a-5p, miR-146a-5p, miR-181a-5p, and miR-574-3p and genes using the miRWalk database and disease ontology module revealed several common targets associated with placental insufficiency, cardiovascular risk factors, cardiovascular and cerebrovascular diseases.

**FIGURE 4:**
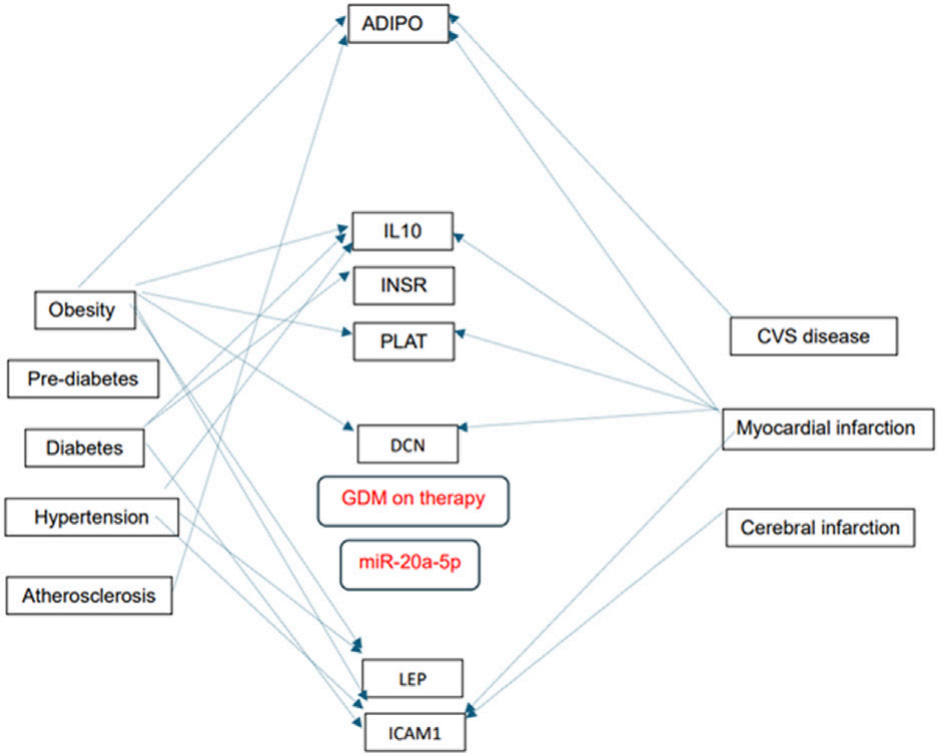
MiRNA-target interactions–Common targets of gestational diabetes mellitus, cardiovascular risk factors, cardiovascular and cerebrovascular diseases. Search for interactions between miR-20a-5p and genes using the miRWalk database and disease ontology module revealed several common targets associated with gestational diabetes mellitus, cardiovascular risk factors, cardiovascular and cerebrovascular diseases.

Numerous predicted and/or validated targets of miRNAs that predict the occurrence of PE have been associated with cardiovascular risk factors and cardiovascular and cerebrovascular diseases (Figures 1A, B). In case of HELLP syndrome, only one common target (CD40LG, the gene encoding the CD40 ligand) associated with cardiovascular risk factors and cardiovascular diseases was identified (Figure 2). Placental insufficiency, usually manifested clinically as preeclampsia and/or fetal growth restriction, has several common miRNA targets associated with cardiovascular risk factors and cardiovascular and cerebrovascular diseases (Figure 3). MiR-20a-5p, a biomarker used solely to predict the occurrence of GDM requiring appropriate therapy, also showed several common targets associated with cardiovascular risk factors and cardiovascular and cerebrovascular diseases (Figure 4).

## 4 Discussion

Currently, no first-trimester predictive algorithm for GH, HELLP syndrome, SGA, GDM, late miscarriage, and stillbirth is available. Novel efficient cost-effective modalities for predicting these pregnancy-related complications at the early gestational stages have been proposed. The proposed approach is based on the combinations of selected maternal clinical characteristics and a minimum number of miRNA biomarkers, which play key roles in cardiovascular system maintenance and control and pathogenesis of cardiovascular diseases and whose altered expression was also observed at early gestational stages in pregnancies with adverse outcomes.

At present, the first-trimester algorithm used by the majority of fetal medicine centres developed by the Fetal Medicine Foundation (FMF) calculates the risks for the development of early PE (before 34 gestational weeks) and FGR (before 37 gestational weeks). The risks are calculated on the basis of knowledge of maternal history, BMI, mean arterial blood pressure (MAP), serum levels of pregnancy-associated plasma protein-A (PAPP-A) and placental growth factor (PIGF), and mean uterine artery pulsatility index (UtA-PI) (O´Gorman et al., 2016; O´Gorman et al., 2017; The Fetal Medicine Foundation, 2023; Tan et al., 2018; Mazer Zumaeta et al., 2020). Using the predictive models based on six miRNA biomarkers and selected maternal clinical characteristics, the detection rate of PE increased 2.50 times and the detection rate of FGR 2.61 times when compared with the first-trimester screening for PE and/or FGR using the FMF algorithm. Moreover, using the proposed approach any subtype of PE and FGR regardless of the severity of the disease (mild and severe PE) and time of disease onset can be detected.

In addition, we demonstrated that numerous predicted and/or validated targets of miRNAs used to predict the occurrence of pregnancy-related complications in the first trimester of gestation were associated with several cardiovascular risk factors and cardiovascular and cerebrovascular diseases.

Pregnancy-related complications have been reported to be associated with the increased risk of later development of diabetes mellitus (Ray et al., 2005; Libby et al., 2007; Lykke et al., 2009; Männistö et al., 2013; Thilaganathan, 2016; Thilaganathan, 2017), metabolic syndrome (Yang et al., 2015; Udenze, 2016), hypertension (Bellamy et al., 2007; Craici et al., 2008; Lykke et al., 2009; Männistö et al., 2013; Hypertension in Pregnancy, 2013; Veerbeek et al., 2015; Thilaganathan, 2016; Thilaganathan, 2017), kidney diseases (Männistö et al., 2013), atherosclerosis (Haukkamaa et al., 2009; McDonald et al., 2013), ischemic heart disease (Irgens et al., 2001; Garovic and Hayman, 2007; Bellamy et al., 2007; Craici et al., 2008; Mongraw-Chaffin et al., 2010; Borna et al., 2012; Berks et al., 2013; Männistö et al., 2013), myocardial infarction (Garovic and Hayman, 2007; Mongraw-Chaffin et al., 2010; Männistö et al., 2013; Hypertension in Pregnancy, 2013; Thilaganathan, 2016; Thilaganathan, 2017), heart failure (Männistö et al., 2013; Hypertension in Pregnancy, 2013; Thilaganathan, 2016; Thilaganathan, 2017), stroke (Irgens et al., 2001; Bellamy et al., 2007; Craici et al., 2008; Mongraw-Chaffin et al., 2010; Berks et al., 2013; Männistö et al., 2013; Hypertension in Pregnancy, 2013; Thilaganathan, 2016; Thilaganathan, 2017) and deep venous thrombosis in mothers (Bellamy et al., 2007; Craici et al., 2008; Lykke et al., 2009).

Based on this evidence, we suggest initiating preventive programs for pregnancies at risk of developing pregnancy-related complications as early as possible with the aim of lowering cardiovascular risk and the consequent development of metabolic, cardiovascular, and cerebrovascular diseases. The dysregulation of miRNAs involving in cardiovascular system maintenance and control may still be reversible via the timely implementation of beneficial lifestyle strategies.

Consecutive large-scale retrospective and prospective analyses are needed to verify the reliability of predictive models based on the combinations of the minimum number of miRNA biomarkers common to adverse pregnancy outcomes and maternal clinical characteristics to differentiate between pregnancies with normal and abnormal courses of gestation at early gestational stages. Gynecologists and obstetricians could have a feasible, cost-effective way of identifying pregnancies at risk of adverse pregnancy outcomes at disposal at early gestational stages if satisfactory discrimination power could be achieved.

The dysregulated miRNAs associated with cardiovascular system maintenance and control may be reversed back to normal via the timely implementation of beneficial lifestyle strategies, which may reduce or delay potential cardiovascular risk in mothers.

## Data Availability

The raw data supporting the conclusions of this article will be made available by the authors, without undue reservation.
